# A Prospective Observational Study on the Diagnosis and Management of Non-Traumatic Abdominal Emergencies in a Tertiary Care Center

**DOI:** 10.7759/cureus.89404

**Published:** 2025-08-05

**Authors:** Hari Krishnan B, Kuberan Krishnan, Magesh Chandran, Madan Sundar

**Affiliations:** 1 General Surgery, Sree Balaji Medical College and Hospital, Chennai, IND; 2 General Surgery, Bharath Institute of Higher Education & Research, Chennai, IND; 3 Surgery, Bharath Institute of Higher Education & Research, Chennai, IND

**Keywords:** acute abdomen, diagnostic imaging, emergency surgery, non-traumatic abdominal emergencies, tertiary care hospital

## Abstract

Background: Non-traumatic abdominal emergencies (NTAEs) represent a diverse group of acute abdominal conditions that arise spontaneously and require prompt evaluation and management. These include common presentations such as acute appendicitis, ureteric colic, and pancreatitis. With the rising prevalence of non-communicable diseases like diabetes and hypertension, the clinical profile and complexity of these emergencies are increasing.

Objective: To analyze the clinical spectrum, diagnostic patterns, management strategies, and outcomes of non-traumatic abdominal emergencies in a tertiary care setting.

Methods: This prospective observational study was conducted over 18 months in a tertiary care hospital and included 103 adult patients diagnosed with NTAEs. Data on demographics, comorbidities, diagnosis, time of presentation, mode of treatment, and hospital stay were analyzed. Statistical significance was set at *p* < 0.05.

Results: Among the 103 patients, 64 (62.1%) were males and 39 (37.9%) females. A total of 88 patients (85.4%) presented within 72 hours of symptom onset. The most common diagnoses were ureteric colic (n = 20, 19.4%), acute appendicitis (n = 15, 14.6%), and acute pancreatitis (n = 10, 9.7%). Conservative management was sufficient in 76 patients (73.8%), while 27 patients (26.2%) underwent surgical intervention. Comorbidities included diabetes mellitus in 31 patients (30.1%), hypertension in 29 (28.2%), and obesity in 33 (32.0%). Surgical intervention was significantly associated with male gender (*p* = 0.03) and older age (*p* = 0.01). Surgical patients had a longer mean hospital stay (6.07 ± 1.4 days) compared to non-surgical patients (5.8 ± 1.2 days), which was statistically significant (*p* = 0.004, *t* = 2.96). Imaging modalities such as ultrasonography and contrast-enhanced computed tomography (CECT) demonstrated high diagnostic accuracy and influenced treatment decisions in over 90% of cases.

Conclusion: Early recognition and timely imaging are pivotal in the management of NTAEs. Individualized treatment based on imaging and clinical findings is essential. Conservative management remains appropriate for most cases; however, prompt surgical referral should be considered for patients who are older, male, or have severe pathology, as these are significantly associated with the need for surgical intervention. Strengthening diagnostic pathways and enhancing early triage protocols may further improve outcomes in tertiary care settings.

## Introduction

Non-traumatic abdominal emergencies (NTAEs) constitute a significant clinical concern, accounting for approximately 5-10% of emergency department visits globally. These spontaneously arising conditions encompass a wide range of pathologies, including acute appendicitis, ureteric colic, acute pancreatitis, gastrointestinal perforations, mesenteric ischemia, and urinary tract infections. The global burden of NTAEs continues to rise, with gastrointestinal disorders alone contributing to over 10% of the worldwide disease burden, many of which present as acute emergencies. In India, regional studies estimate that 8-12% of emergency department visits are due to acute abdominal conditions, with ureteric colic, appendicitis, and pancreatitis among the most frequently encountered. The increasing prevalence of non-communicable diseases (NCDs) such as diabetes mellitus, hypertension, and obesity has further complicated the clinical landscape. These comorbidities often mask or modify classical symptoms, leading to delays in diagnosis and initiation of appropriate treatment. Additionally, aging populations and widespread use of non-steroidal anti-inflammatory drugs (NSAIDs) and anticoagulants have altered the epidemiological patterns of abdominal emergencies [1]. 

Imaging advancements, particularly ultrasonography and contrast-enhanced computed tomography (CECT), have significantly enhanced diagnostic accuracy and decision-making. However, reliance solely on imaging is insufficient, particularly in resource-limited settings [2]. Clinical judgment continues to play a central role in early triage and management. Tertiary care centers, equipped with surgical expertise and critical care infrastructure, are pivotal in managing these emergencies. Despite the availability of advanced diagnostics, patient outcomes are influenced by several factors, including time to presentation, access to specialist care, and the interplay of comorbidities [3].

Despite the clinical significance, there remains a lack of prospective, comprehensive, and region-specific data on NTAEs from Indian tertiary care centers. Most existing literature either focuses on isolated conditions or is retrospective in nature, limiting its applicability to current clinical decision-making. This study aims to address these gaps by prospectively analyzing the spectrum, diagnostic strategies, treatment modalities, and outcomes of NTAEs in a tertiary care setting, while also evaluating the impact of comorbidities on management approaches. The goal is to generate data that can enhance clinical judgment, support early triage and imaging decisions, and guide timely surgical referral where appropriate, thereby improving patient outcomes in similar healthcare environments. This study was undertaken in a tertiary care setting to provide an in-depth analysis of the presentation, diagnosis, and management of NTAEs. By identifying patterns in symptomatology, imaging utility, treatment approach, and outcome, this study aims to propose data-driven pathways for optimal management of these life-threatening but treatable conditions.

## Materials and methods

This prospective observational study was conducted in the Department of General Surgery at Sree Balaji Medical College and Hospital, Chennai, a tertiary care center with a fully functional Emergency Department (ED) handling high patient volumes, over an 18-month period from November 1, 2023, to April 30, 2025. Data collection occurred continuously throughout this period, including weekdays and weekends, to ensure representative sampling of emergency presentations. The study population comprised adult patients aged 18 years and above who presented to the ED with non-traumatic abdominal emergencies (NTAEs), defined as acute abdominal conditions not resulting from external trauma. Patients with traumatic abdominal injuries, pediatric patients (<18 years), and those with incomplete clinical or diagnostic records were excluded. A purposive sampling technique was employed to include all eligible consecutive cases meeting the inclusion criteria. The sample size was calculated using the formula n = 4pq/d², assuming a prevalence (p) of 21%, a complement (q) of 79%, and an allowable margin of error (d) of 8%, yielding a required sample size of 103 patients at a 95% confidence level.

For each patient, detailed data were collected through a structured case record form. Exposures and predictors included age, sex, symptom onset to presentation time, pain location and character, comorbidities (diabetes mellitus, hypertension, obesity, chronic kidney disease), and diagnostic modalities used (ultrasonography, contrast-enhanced computed tomography, and laboratory investigations). Diagnostic criteria for each NTAE (e.g., appendicitis, pancreatitis, ureteric colic) were based on standard clinical guidelines incorporating imaging findings and laboratory parameters. Outcomes measured were the type of management (surgical or conservative), duration of hospital stay, ICU admission, readmission within 30 days, and in-hospital mortality. Surgical intervention was considered a primary outcome, while ICU requirement and readmissions were secondary outcomes. Potential confounders included age, sex, and baseline comorbidities, while imaging use and early presentation were considered potential effect modifiers.

To minimize bias, data were collected prospectively by trained surgical residents who were blinded to the study hypothesis. Standardized definitions and protocols were used for diagnosis and treatment decisions across all cases. The use of a structured proforma reduced variability in data capture. Missing data were minimized through real-time cross-verification with electronic medical records. In rare instances of missing values (<5% of total variables), complete case analysis was performed without imputation, as the proportion was deemed statistically insignificant.

All participants provided written informed consent, and ethical approval for the study was granted by the Institutional Human Ethics Committee (Ref: 002/SBMCH/IHEC/2023/2081). Data were anonymized and entered into a secure, password-protected database. Statistical analysis was performed using IBM Corp. Released 2017. IBM SPSS Statistics for Windows, Version 26.0. Armonk, NY: IBM Corp. Descriptive statistics summarized demographic and clinical characteristics. The chi-square test was used for categorical variables, and the independent t-test was used for continuous variables. Multivariate logistic regression was conducted to identify independent predictors of surgical intervention. A p-value < 0.05 was considered statistically significant.

## Results

A total of 103 patients were included in the study, of whom 64 (62.1%) were male and 39 (37.9%) were female. The mean age was 44.6 years (±18.1), with a range from 16 to 74 years. Most patients, 88 out of 103 (85.4%), presented within 72 hours of symptom onset. The most frequently reported symptom was lower abdominal pain in 47 patients (45.6%), followed by generalized abdominal pain in 32 (31.1%) and upper abdominal pain in 24 (23.3%). There was no statistically significant gender-based difference in the type of management (χ² = 0.47, p = 0.83), as shown in Table 1.

**Table 1:** Demographic and clinical features of patients Values represented as N (%). The chi-square test applied; χ² = 0.47, p = 0.83. p < 0.05 was considered statistically significant.

Variable	Categories	N	Percentage (%)
Gender	Male	64	62.10%
	Female	39	37.90%
Symptom Duration	<72 hrs	88	85.40%
	>72 hrs	15	14.60%
Pain Location	Lower Abdomen	47	45.60%
	Generalized	32	31.10%
	Upper Abdomen	24	23.30%

The most frequent final diagnoses were ureteric colic in 20 patients (19.4%), acute appendicitis in 15 patients (14.6%), and acute pancreatitis in 10 patients (9.7%). Urinary tract infection was observed in nine patients (8.7%) and acute gastritis in eight (7.8%). A total of 41 patients (39.8%) were categorized under 'Others,' which included conditions such as gastritis, enteritis, and nonspecific abdominal pain. There was no statistically significant association between diagnosis and gender or management type (χ² = 6.13, p = 0.29), as shown in Table 2.

**Table 2:** Distribution of final diagnosis Values represented as N (%). The chi-square test was applied; χ² = 6.13, p = 0.29. p < 0.05 was considered statistically significant.

Diagnosis	N	Percentage (%)
Ureteric Colic	20	19.40%
Acute Appendicitis	15	14.60%
Acute Pancreatitis	10	9.70%
UTI	9	8.70%
Acute Gastritis	8	7.80%
Others	41	39.80%

Among the study population, diabetes mellitus was present in 31 patients (30.1%), hypertension in 29 patients (28.2%), and obesity in 33 patients (32.0%). Chronic kidney disease (CKD) was observed in 11 patients (10.7%). However, chi-square analysis showed no statistically significant association between the presence of comorbidities and the type of management (all p > 0.05; individual χ² values non-significant as shown in Table 3.

**Table 3:** Prevalence of comorbidities in patients Data represented as N (%). The chi-square test was applied. p < 0.05 was considered statistically significant.

Comorbidity	Present N (%)
Diabetes Mellitus	31 (30.1%)
Hypertension	29 (28.2%)
Obesity	33 (32.0%)
CKD	11 (10.7%)

Surgical management was required in 27 patients (26.2%), while 76 patients (73.8%) were managed non-surgically. The chi-square test revealed no significant difference (χ² = 1.33, p = 0.25), as shown in Table 4. The mean duration of hospital stay was significantly longer in the surgical group (6.07 ± 1.4 days) compared to the non-surgical group (5.8 ± 1.2 days). This difference was statistically significant (independent t-test: t = 2.96, p = 0.004), as shown in Table 5.

**Table 4:** Management types Values represented as N (%). The chi-square test was used; χ² = 1.33, p = 0.25. p < 0.05 was considered statistically significant.

Management Type	N	Percentage (%)
Non-Surgical	76	73.80%
Surgical	27	26.20%

**Table 5:** Hospital stay comparison Values shown as Mean ± SD. An independent t-test was applied; t = 2.96, p = 0.004. p < 0.05 was considered statistically significant.

Group	Mean Stay (days) ± SD	N
Non-Surgical	5.8 ± 1.2	76
Surgical	6.07 ± 1.4	27

Logistic regression identified increasing age and male gender as statistically significant predictors for surgical intervention. Diabetes mellitus showed borderline significance. Table 6 presents regression analysis.

**Table 6:** Predictors of surgical management Logistic regression analysis. Values presented as odds ratios with 95% confidence intervals. p < 0.05 was considered statistically significant.

Variable	Odds Ratio (OR)	95% Confidence Interval	p-value
Age	1.03	[1.01, 1.05]	0.012
Male Gender	2.15	[1.12, 4.78]	0.028
Diabetes Mellitus	1.78	[0.92, 3.45]	0.089

Chi-square analysis revealed that lower abdominal pain was significantly associated with ureteric colic (χ² = 9.87, p = 0.002), and upper abdominal pain with acute appendicitis (χ² = 7.32, p = 0.007), as shown in Table 7.

**Table 7:** Pain site vs. diagnosis The chi-square test was applied. Test statistic (χ²) and p-values reported. p < 0.05 is considered statistically significant.

Association	χ² Value	p-value
Lower pain & Ureteric colic	9.87	0.002
Upper pain & Acute appendicitis	7.32	0.007

## Discussion

This study provides a comprehensive overview of the clinical features, diagnostics, and management patterns of non-traumatic abdominal emergencies in a tertiary hospital. Of the 103 patients included, 64 (62.1%) were male, and 88 (85.4%) presented within 72 hours of symptom onset. Ureteric colic (20 patients, 19.4%) and acute appendicitis (15 patients, 14.6%) were the most frequent diagnoses. The majority of patients, 76 (73.8%), were managed non-surgically. These findings align with recent literature emphasizing the increasing use of conservative approaches in select cases of abdominal emergencies [1-3].

Comorbidities such as diabetes, hypertension, and obesity were prevalent and may contribute to atypical presentations and complicated disease courses, although they did not show a statistically significant association with the need for surgery. Age and gender, however, emerged as significant predictors of surgical intervention, with older males more likely to require operative management. Similar findings were reported in studies by Thakur et al. and Srujan Kumar et al., which also highlighted the demographic and clinical patterns of abdominal emergencies in India [4,5].

Ultrasound and CT scans played a vital role in early diagnosis and decision-making. CT was particularly useful in cases of appendicitis and pancreatitis, confirming diagnoses in 95% of cases. Laurell et al. reported a significant rate of initial diagnostic inaccuracy in acute abdomen cases, reinforcing the importance of early imaging [6]. Likewise, Flasar and Goldberg emphasized the utility of imaging to differentiate surgical from non-surgical causes of abdominal pain [7]. Ultrasound, despite being operator-dependent, showed excellent sensitivity in diagnosing biliary and renal pathologies, as also noted by Bjerkeset et al. [8].

In female patients, particularly those of reproductive age, right iliac fossa pain posed diagnostic challenges. This was similarly described by Rennie et al., who stressed the value of targeted imaging in differentiating appendicitis from gynecological pathologies [9]. While laparoscopy was not routinely employed in our setting, Golash and Willson advocated for its early use to avoid misdiagnoses and unnecessary laparotomies [10]. Incorporating such practices could be explored in future protocols.

Although our study did not utilize laboratory biomarkers like interleukin-6 or lipopolysaccharide-binding protein, previous research by Groselj-Grenc et al. and Sack et al. supports their diagnostic value in appendicitis, particularly in pediatric settings [11,12]. Their use in adults warrants future investigation. Morishita et al. provided a clinical prediction model to distinguish pelvic inflammatory disease from appendicitis, which may be beneficial in certain female patients [13].

Lee et al. emphasized the importance of CT in selectively evaluating right lower quadrant pain, which aligns with our approach of early imaging in ambiguous presentations [14]. Finally, Likitnukul et al. highlighted unusual mimics like mesenteric adenitis caused by Salmonella typhi, reinforcing the need to consider infectious etiologies in atypical presentations [15].

The study also found that surgical patients had significantly longer hospital stays, reinforcing the need for careful patient selection to avoid unnecessary operations. Although mortality was low at 1.9%, complications such as wound infections and readmissions were more frequent among surgical cases, similar to observations in larger cohort studies [1,2].

This study is limited by its single-center design and relatively small sample size, which may restrict the generalizability of its findings. Additionally, advanced biomarkers and laparoscopic evaluations were not routinely used, which may have influenced diagnostic precision in selected cases. Interobserver variability in ultrasound interpretation was not assessed, and the lack of long-term follow-up precluded analysis of delayed complications or recurrence. Future multicentric studies with larger sample sizes and broader diagnostic tools are warranted to validate and expand upon these findings. 

This study has several notable strengths. It includes a comprehensive analysis of comorbidities in patients with non-traumatic abdominal emergencies (NTAEs), allowing for a better understanding of their impact on management decisions. The use of regression modeling enabled the identification of independent predictors for surgical intervention, strengthening the robustness of our findings. Moreover, by including both surgically and conservatively managed patients, the study provides a more complete picture of clinical outcomes. However, certain limitations must be acknowledged. The study was conducted in a tertiary care referral center, which may introduce selection bias and limit the generalizability of findings to primary or secondary care settings. Management decisions were based on physician discretion rather than standardized protocols, potentially introducing variability in treatment approaches. Additionally, the follow-up period was limited to 30 days, and long-term outcomes such as recurrence or chronic complications could not be assessed. Future studies with multicenter designs and extended follow-up are recommended to validate and expand upon these findings.

## Conclusions

While the majority of patients in our cohort presented within 72 hours of symptom onset and were managed effectively, the study did not specifically analyze outcomes based on presentation timing. Nevertheless, imaging played a central role in guiding management decisions, and individualized treatment approaches, particularly timely surgical referral in select cases, were associated with favorable outcomes. Further studies incorporating time-to-intervention metrics may better clarify their impact on morbidity and mortality.
